# Preliminary Capsulotomy in the Extraction of Cataract

**Published:** 1890-11

**Authors:** T. J. Tyner

**Affiliations:** Austin, Texas


DANIEL'S

### TEXAS MEDICAL JOURNAL.

A Representative Organ of the Medical Profession, and an Exponent of Rational Medicine; devoted to the Organization, Advancement and Elevation of the Profession in Texas.

PUBLISHED MONTHLY.—SUBSCRIPTION $2.00 A YEAR.

Vol. VI. AUSTIN, NOVEMBER, 1890. No. 5.

### Original Articles.

CONTRIBUTED EXCLUSIVELY TO THIS JOURNAL.

*The Articles in this Department are accepted and published with the understanding that we are not responsible for, nor do we indorse the views and opinions of the writers by so doing.*

### PRELIMINARY CAPSULOTOMY IN THE EXTRACTION OF CATARACT.

BY T. J. TYNER, M. D., AUSTIN, TEXAS.

[Prepared for the International Medical Congress.—ED.]

OWING to the great amount of literature recently devoted to the subject of cataract extraction, I owe it to you as a matter of courtesy, as well as in justice to myself, to say I would not presume to bring it forward now had I not failed after diligent search to find a precedent for the operation which I shall hereafter describe, and which I believe possesses some merit. The nearest approach to it is in opening the capsule with the point of the knife, as it enters the anterior chamber while the section is being made, and with which you are all familiar.

The leading point in the operation is in making the capsulotomy the primary step, thereby enabling the operator to deliver the lens at the very moment the corneal section is completed. I will not encroach upon your time with the progressive history of the many methods devised by different operators, nor with the detail of this operation as to instruments, antiseptics, after-treatment, etc., as they differ in no essential particular from the generally accpted measures in other methods.

Supposing the eye to be now ready. A Bowman stop-needle is thrust into the anterior chamber—the pupil having been previously dilated—the point of which, and also the entire field of the incision, are in full view.

The capsule is now lacerated in its upper quadrant, the line of incision corresponding to the upper pupillary curve of the iris. In this manipulation and in withdrawing the needle, the greatest care should be observed that no aqueous is lost. The eye is now practically undisturbed and as favorable for the corneal section as before, which is to be done quickly, using a Græfe knife, preferably rather broad. When the section is finished, pressure with the flat of the blade causes the corneal opening to gape, when at the same moment counter-pressure with the fixing forceps below aids the expulsion and the lens glides out through the still open pupil with surprising ease.

I will mention here that the lens, having no choice, or rather no other avenue of escape, almost always indicates a tendency to follow the knife as the corneal incision is progressing, and when it is finished the lens is partly in the anterior chamber. I state this to demonstrate why it is so promptly delivered, and that the foregoing expression is not extravagant.

The operation is simple throughout and easily done, and is accomplished when the most difficult part in other methods begins. An additional point of interest is: If the lens is susceptible of being dislocated—and this is made manifest as soon as the needle touches the capsule—there is, in my experience, no way to accomplish it so perfectly and harmlessly as with the needle at this stage of the operation. This is somewhat similar to Delgardo's method, and, strange to say, was the result in my first case, which occurred last October. Since then I have performed the operation twelve times with a good result in each one, or, to be more definite, with the exception of two cases, the result was far better than that formerly achieved. In the two cases referred to there was severe iritis with posterior synechia, and in four others it was manifest, but only in a mild form. In the remaining six cases there was absolutely no reaction. I am inclined to think the iritis was in part due to the excessive strength of the atropine used in dilating the pupil, which, a few hours after the operation, reasserts itself, hence crowding the iris nearer the corneal wound. I now use the weakest solution of atropine that will serve the purpose. Eserine might be useful in some cases, though as yet I have not felt the necessity of resorting to it.

I neglected to mention in the foregoing statement that in three of the foregoing cases the lenses were extracted in their capsules.

If you will now bear with me a few moments longer, and I trust not without interest, I will relate the circumstances, which by the way were partly accidental, that led up to the development of the operative procedure above described. In July, 1885, I operated on a Mexican, and while I was opening the capsule, having done an iridectomy, fluid vitreous escaped so rapidly that the globe was so collapsed that the lens could only be delivered by the aid of the iris forceps, having fallen into the posterior chamber. Singular to say, there was a good recovery with useful vision, which result encouraged me a few weeks later to attempt the extraction in the other eye. Anticipating the same condition of vitreous, the thought suggested itself to open the capsule with a needle previous to making the corneal section. This was successfully performed, and, while there was loss o vitreous (fluid), it was slight compared to the first. This case is recorded in the published statistics of Texas surgery in 1886. This little procedure passed out of my mind until the discussion became so general in regard to a return to the simple extraction, which later on was adopted by most operators. It was not my wish to give up the iridectomy, but in the mean time, however, I had several cases in which the lens popped out through the pupil just as the section was completed—one in which I had opened the capsule with the point of the knife as it entered the anterior chamber, the patient at the moment the section was finished squeezing the eye. Another case was traumatic, in which the particle of steel could be distinctly seen in the lens, and had thoroughly lacerated the capsule. This was a fac-simile of the preceding case, the fragment of steel coming with the lens. This case, together with others, impressed upon my mind that the lens indicated a tendency to escape, and, as a consequence, sought the course of least resistance. Upon this hypothesis I endeavored to make the simple extraction in this way—*i. e.*, by opening the capsule with the point of the knife; but it was attended with so many failures to make the rapid extraction without injury to the iris that I abandoned it. About this time I recalled to mind the preliminary capsulotomy done with the needle in 1885, and a few months later (after returning from my summer vacation) I put it into practice with the results above given.

